# Women's evaluation of abuse and violence care in general practice: a cluster randomised controlled trial (weave)

**DOI:** 10.1186/1471-2458-10-2

**Published:** 2010-01-02

**Authors:** Kelsey L Hegarty, Jane M Gunn, Lorna J O'Doherty, Angela Taft, Patty Chondros, Gene Feder, Jill Astbury, Stephanie Brown

**Affiliations:** 1 Department of General Practice, University of Melbourne 200 Berkeley St, Carlton, Melbourne, Australia

## Abstract

**Background:**

Intimate partner abuse (IPA) is a major public health problem with serious implications for the physical and psychosocial wellbeing of women, particularly women of child-bearing age. It is a common, hidden problem in general practice and has been under-researched in this setting. Opportunities for early intervention and support in primary care need to be investigated given the frequency of contact women have with general practice. Despite the high prevalence and health consequences of abuse, there is insufficient evidence for screening in primary care settings. Furthermore, there is little rigorous evidence to guide general practitioners (GPs) in responding to women identified as experiencing partner abuse. This paper describes the design of a trial of a general practice-based intervention consisting of screening for fear of partner with feedback to GPs, training for GPs, brief counselling for women and minimal practice organisational change. It examines the effect on women's quality of life, mental health and safety behaviours.

**Methods/Design:**

**weave **is a cluster randomised controlled trial involving 40 general practices in Victoria, Australia. Approximately 500 women (16-50 years) seen by the GP in the previous year are mailed a short lifestyle survey containing an item to screen for IPA. Women who indicate that they were afraid of a partner/ex-partner in the last year and provide contact details are invited to participate. Once baseline data are collected, GPs are randomly assigned to either a group involving healthy relationship and responding to IPA training plus inviting women for up to 6 sessions of counselling or to a group involving basic education and usual care for women. Outcomes will be evaluated by postal survey at 6 and 12 months following delivery of the intervention. There will be an economic evaluation, and process evaluation involving interviews with women and GPs, to inform understanding about implementation and outcomes.

**Discussion:**

The **weave **trial responds to an urgent need for more evidence on what can be achieved in primary care with regard to responding to women who experience IPA. It will provide important knowledge about the effectiveness of a brief method of screening, professional IPA training program and brief counselling for women.

**Trail Registration:**

[ACTRN12608000032358]

## Background

Intimate partner abuse (IPA) or violence is defined as any behaviour within an intimate relationship that causes physical, psychological or sexual harm to those in the relationship [1]. Behaviours include acts of physical aggression such as slapping and kicking; psychological abuse such as intimidation and humiliation; forced intercourse and other forms of sexual coercion; and various controlling behaviours such as isolating a person from their family and friends, monitoring their movements, and restricting access to information or assistance. IPA sits within the broader context of gendered violence and the majority of assaults by partners are directed at females [1,2]. Moreover, sexual abuse and partner violence resulting in significant injury are much more commonly perpetrated against women by their partners than against men [3]. Partner abuse is a major public health problem globally. It diminishes women's capacity to participate in occupational, social and familial life and contributes to significant morbidity and mortality among women of child-bearing age [4]. IPA is a complex problem arising from an interplay of personal, situational and socio-cultural factors [2]. Thus, in addition to the need for multifaceted social and educational interventions, early intervention in healthcare settings is required. Primary care offers such an opportunity.

### Prevalence of IPA

Partner abuse is a common but hidden problem for women of child-bearing age. Across ten culturally and economically diverse countries, the World Health Organisation reported the lifetime prevalence of physical and/or sexual partner violence as ranging from 15% to 71% [5]. An Australian general practice study found that almost 30% of women had at some point in their lives been afraid of a partner [6]. A further GP study using the Composite Abuse Scale (CAS) [7] to measure abuse in the previous 12 months reported that 6% of women of child-bearing age had experienced severe combined physical, emotional and/or sexual abuse; a further 7% experienced physical and emotional abuse; 6% experienced physical abuse alone and 6% reported emotional abuse alone [8]. Similarly, a United Kingdom study reported that 17% of women attending general practice had experienced physical violence from a partner/ex-partner in the previous year [9].

### Health consequences of IPA

Partner abuse has been estimated as the leading cause of death and disability among women of child-bearing age [10]. Research consistently highlights a range of severe physical and mental health problems that are associated with partner abuse [4]. Abused women are at increased risk of anxiety, depression, post-traumatic stress disorder, suicide, and drug and alcohol abuse [8,11,12]. Women indicate that the psychological abuse is even more difficult to endure than the physical abuse itself [13]. The most common physical health problems include injuries, chronic pain and gynaecological, cardiovascular, neurological and gastrointestinal problems [14]. Partner abuse may commence, or increase during pregnancy affecting up to 1 in 4 pregnant women [13,15]. In a UK cross-sectional study of women attending general practice, 15% of respondents who had ever been pregnant reported partner violence during pregnancy, with a quarter reporting that this violence was worse than when they were not pregnant and almost one third saying that it had caused a miscarriage [9]. Partner abuse is associated with adverse maternal and infant outcomes e.g. low birth weight [16], foetal injury and pre-term birth [17], and even death of the mother or the foetus [18]. Partner abuse also has associations with common maternal physical health issues - back pain, headache, urinary incontinence and some less common health issues such as bleeding in first trimester, faecal incontinence [19,20]. Partner abuse also has serious consequences for the physical and emotional well-being of children who witness it [21].

### IPA and health care

Abused women are overrepresented in outpatient settings and in primary care [22,23]. Approximately a third of abused women disclose abuse to their GP [24]. Women describe barriers to disclosure that are both internal (e.g. feeling ashamed and embarrassed) and external (e.g. perceiving that doctor is only there for physical problems). GP inquiry is associated with increased disclosure [24], however only 1 in 10 abused women are asked about abuse by the GP [24,25]. Yet there is evidence that women consider it appropriate to be asked about partner abuse [26]. This is moderated by the context of the consultation, the relationship with the health care provider and the woman's readiness to address the problem [27]. Reluctance on the part of health professionals, including GPs, to inquire about abuse owes to factors such as lack of time and training, lack of effective interventions and the complexities of providing whole family care [28,29]. Low levels of inquiry and disclosure have triggered a shift in research focus from studies about prevalence, consequences and patient-health provider interactions to finding improved approaches to screening and intervention.

### Screening

A recent systematic review shows that there is insufficient evidence to justify implementing screening programs [30]. Further support came from a recent Canadian study [31], the first IPA screening trial to examine health outcomes for women. It included 12 primary care sites. The authors concluded that there was not enough evidence to support IPA screening in health care settings as routinely asking all patients in the intervention group about abuse, though not found to be harmful, was no more beneficial in terms of health outcomes than usual care. There was no specific intervention offered to women who were detected by the screening program. Despite women's doctors being informed that they screened positive, half reported that IPA was not raised in subsequent consultations. A major criterion for screening that is not being met relates to the availability of an effective treatment once abuse is identified/disclosed. This means that IPA fails to fulfil public health policy criteria for a screening program in health care settings [30]. There is therefore an urgent need for rigorous testing of specific interventions and services for women following identification of IPA [31,32]. IPA screening instruments are increasingly evaluated against criterion standards such as the Conflict Tactics Scale [33] or Composite Abuse Scale [7]. In a review of 18 brief screening tools in 15 validation studies, Feder et al. found several to be valid for use in health care settings [30]. Inquiring about fear of a partner or ex-partner is receiving increased attention [6,34] and has significant potential as a stand alone screening item. Abused women attending primary care are much more likely (OR = 64.1, 95% CI 44.4-94.1) to have been afraid of a partner or ex-partner at some point in their lives than non-abused women [6]. The fear question has been shown to have good sensitivity and specificity for identifying women who have experienced physical abuse (75.5% sensitivity, 82.4% specificity) or severe combined physical, emotional and sexual abuse (85% sensitivity, 77.7% specificity) in a large sample of women attending GPs for primary care. It does not perform as well in identifying women who have experienced emotional abuse alone (60.6% sensitivity, 80.4% specificity) [7]. It may be concluded that the implementation of screening for IPA is hampered by the absence of evidence for intervention following screening, particularly intervention for women in the early stages of recognising and disclosing abuse. Therefore expanding the evidence base on the optimal method of screening and effective responding is a priority.

### Interventions for women in health care settings

Ramsay et al. reviewed 19 studies to evaluate the effectiveness of health care interventions for women on physical and psychosocial wellbeing and their experiences of abuse [35]. This was recently updated with the addition of 14 studies, 5 of which focused on children for the first time [30]. Studies came from diverse settings (e.g. antenatal clinics, refuges, community settings, primary care) and variously tested the impact of advocacy, support group and psychological (individual or group) interventions on outcomes such as post-traumatic stress disorder, depression, self-esteem and abuse. Overall, the evidence was sufficient to recommend access to advocacy services but this only applied to women who had actively sought help (as opposed to women identified through screening). Evidence for the effectiveness of psychological group therapy, support groups, and child interventions was insufficient on account of too few studies, poor quality design and lack of data for calculating effect sizes. There was sufficient evidence to recommend individual psychological treatments. However, treatments were diverse (e.g. cognitive behavioural therapy, problem-solving, expressive writing, psycho-education, feminist-oriented and grief counselling and forgiveness therapy) and since they largely involved survivors and those actively seeking assistance, they can be extrapolated neither to women identified through screening nor those attending primary care settings. There was a clear absence of qualitative studies examining what women themselves think should be contained in an intervention for IPA [30].

Similar to the absence of women's voices, primary care was under-represented across these studies and settings. The review demonstrates the lack of focus on early intervention and the need for more evidence about woman-centred interventions. While health practitioners are widely encouraged to assume a role in supporting abused women, there are limited guidelines available on how to do this [36]. Most tend to focus on identification and referral rather than on appropriate ways of responding to and counselling women following disclosure. It is imperative to expand the evidence base with respect to the types of counselling that might be effective for abused women who screen positive for abuse. This paper describes the development and design of a trial of screening and intervention in primary care for women who have been afraid of a partner or ex-partner in the last year.

### Evidence informing the development and design of weave

We have outlined in detail [37] the development of the counselling intervention based on the Transtheoretical Model of Behaviour Change [38] adapted to partner abuse [39,40]. We particularly focused on the 'Psychosocial Readiness Model' [41] to conceptualise women's experiences. We used evidence of best practice from systematic reviews of health care-based interventions [30,35] and of qualitative studies with women [27], international primary care guidelines on partner violence [36] and evaluation of general practice-based partner abuse pilots in Australia and overseas. The **weave **brief counselling intervention [37] incorporates motivational interviewing [42] and problem-solving techniques [43], which have been increasingly applied in the primary care setting for depression issues [44]. Finally, partner abuse interventions frequently aim to improve the safety of women [45-47], and this forms a core aspect of our 'Healthy Relationships' training for GPs.

### Outcomes

A key issue in trial design is to identify a set of outcomes that are important to women experiencing abuse and selecting an appropriate means of operationalising these outcomes. Programs focused on women should not be expected to necessarily produce decreased violence in women's lives [48] suggesting that the use of violent events as a primary outcome in trials may be problematic. Change that is internal to the woman is potentially more informative when evaluating the impact of an intervention for partner abuse, especially one that involves direct counselling. Indeed it may be that significant changes in experience of abuse may not be observable for some time after the seeds of change have been sown. Instead it may be more effective to focus on health outcomes for women, such as quality of life and mental health, which have received limited attention in trials to date [31,49]. Emergent areas of measurement include harm - that which potentially emanates from screening, intervention and from participating in IPA research [30].

### Aims of weave

The primary aim of the **weave **study is to determine if a multifaceted intervention consisting of screening for intimate partner abuse and feedback for GPs, training for GPs, a brief counselling intervention for women and minimal practice organisational change results in:

• increased quality of life;

• increased mental health, and;

• increased safety behaviours and planning for women who experience partner abuse.

The secondary aims are to determine if the intervention results in:

• increased readiness for change with regard to the abuse;

• increased comfort on the part of women to discuss partner abuse with GPs;

• increased inquiry by GPs about the safety of women and children;

• reduced anxiety and depression;

and is cost effective.

We hypothesise that the brief counselling intervention will increase women's perceived support and comfort to discuss abuse and lead to positive changes in women's self-efficacy and readiness to change, and that these 'internal' changes will collectively lead to increases in safety planning and behaviours and improvement in mental health and quality of life.

## Methods/Design

**Figure 1:**
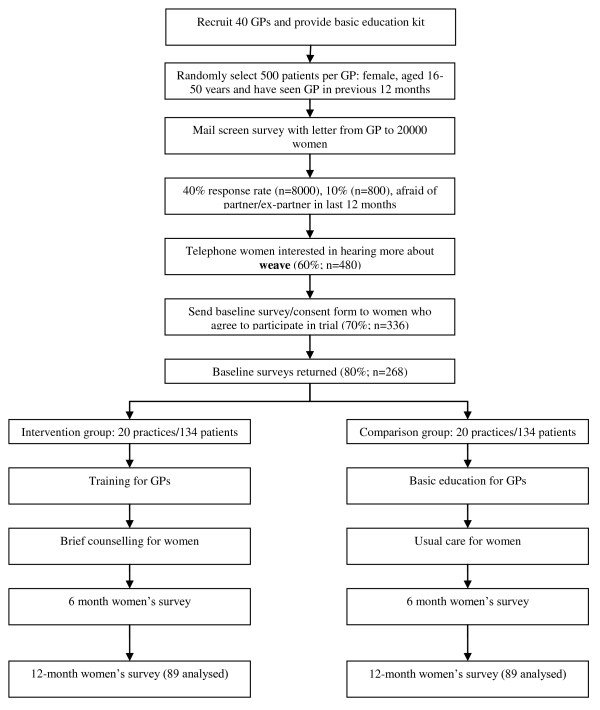
**Flow of participants through trial**.

The study conforms to the guidelines contained in the CONSORT statement for cluster randomised controlled trials [50]. Individual GPs (cluster) will be the unit of randomisation. The study includes one GP only per practice to circumvent the threat of contamination due to cross-over effects. Interventions and analyses will target two levels - the cluster (GP) level and individual (woman) level. The trial will include 40 GPs and consist of two arms - intervention and comparison. Figure 1 presents the anticipated flow of clusters and individual patients over the course of the trial. The study has received ethics approval from the Human Research Ethics Committee of The University of Melbourne.

### Inclusion and exclusion criteria

#### General practitioners

GPs will be eligible if they work three or more sessions per week and are based at a computerised practice. GPs will be excluded if 30% or more of their patients are non-English speaking or if the GP has not been actively practising in the last 12 months.

#### Women

Women will be eligible for the initial screening component of the study if they have consulted the participating GP within the last 12 months and are aged between 16 and 50 years. Women will be excluded if, in exercising clinical judgement, the GP anticipates they may encounter difficulties in providing informed consent, understanding the content of surveys and/or participating in other aspects of the study due to mental or physical health issues, cognitive impairment, intellectual disability or poor English language skills. Additional criteria are required for inclusion in the trial - women will be invited to participate (over the telephone) if they indicate in the screening survey that they have been afraid of their partner or ex-partner in the last 12 months and are interested in hearing more about the **weave **project. Women will be excluded at this stage if it is established during the recruitment phone call that they no longer attend the GP or they are a false-positive. False positives are women who misinterpreted the fear item in that they have never felt afraid of a partner or they have not felt afraid in the previous 12 months.

### Number of participants required

The final sample size of 89 women in each of the two groups will have at least 80% power (alpha 5%, 2-sided test) allowing for a clustering effect (intra-cluster correlation of 0.02 [8]) to detect clinically important differences on the primary outcomes at 12 months between the intervention and comparison groups (See Table 1). To have sufficient power to test our hypotheses, 40 practitioners (20 in each arm) are required in order to allow screening of approximately 500 women per practice (20000 women in total). Based on women's response rates from the **weave **pilot study and *diamond *study [51] 40% of women will return the screening survey (8000). Of these, it is estimated that 10% (800) will have experienced abuse that includes combined physical, sexual and/or emotional abuse in the last 12 months and will therefore screen positive to having been afraid of a partner or ex-partner during this period. Of these, 60% (480) will indicate an interest in hearing more about the project and being contacted by the research team. It is estimated that 70% (336) of these women will agree over the phone to being involved in the trial (a proportion will decline and a proportion will prove ineligible at this stage), 80% (268) of whom will return their baseline surveys and enter the trial. Following randomisation, approximately a third (88) will be lost to follow-up at 12 months based on data from the *diamond *cohort [51] and MOSAIC [52] leaving 89 women per group at 12 months.

**Table 1:** Primary outcomes, measures and hypothesised differences between study groups

Outcome	Measure/tool	Hypothesis
Quality of life	World Health Organisation Quality of Life-Bref [58]	There will be a difference of half of a standard deviation between the two groups (assuming a SD of 20) [66]
Mental health status	SF-12 Mental Component Summary [59]	There will be a difference of half of a standard deviation (SD = 11) between the two groups [67]

Safety planning	Safety plan in the last 12 months	Have a safety plan at 12 months: 10% vs 40%
Safety behaviours	Safety-Promoting Behaviour Checklist [60]	There will be a difference of half of a standard deviation (SD= 2.5) between the two groups [68]

### Recruitment

Multiple strategies will be used to recruit GPs. These include mailing to randomly selected GPs (750 urban, 250 rural; within 150 km radius of Melbourne) registered with the Australasian Medical Publishing Company. GPs will be sent a letter of invitation, information about the project and a faxback form in the mail. If we still require more GPs, we will re-contact eligible practices from this original list, and request that the practice manager advertise the project among GPs with interest in women's health, domestic violence, mental health or research. Additionally, we will mail out to 600 GPs involved in shared maternity care in Melbourne using the same protocol.

Although we will utilise the lists described above as much as possible to minimise selection bias, we will enlist the services of VicReN if required. This is a Victorian-based general practice research network service based at the Primary Care Research Unit at The University of Melbourne. Staff from VicReN will assist by advertising the project in newsletters of the Royal Australian College of General Practitioners (RACGP) and Divisions of General Practice and by engaging GPs using various strategies. All eligible GPs will be asked to read and sign a Memorandum of Understanding and consent form, complete a baseline survey (allowing comparison with the Australian GP population) and to complete the basic education kit. Practices are reimbursed at a rate of $500 for time involved in generating patient lists and GPs will be eligible for RACGP Quality Assurance and Continuing Professional Development points.

### Patient recruitment

Patient recruitment will be done through methods validated in a recent primary care cohort study that screened for probable depression via postal survey, and included a screen for abuse [51]. In **weave**, for each participating GP, a list of female patients, aged 16 to 50 years who consulted the GP at least once in the previous 12 months will be randomly generated (maximum 600 patients per list). The GP will review the list and exclude those women who meet exclusion criteria. The remaining women will be mailed the screening questionnaire together with a letter from their GP endorsing the project, an information sheet, a resource card listing contact numbers for various support agencies and a reply paid envelope. In the survey the respondents are told that the **weave **team is trying to work out ways to improve the care women receive in general practice, and particularly in relation to emotional well-being. At the end of the screening survey, respondents are asked if they would like to hear more about **weave**, the next stage of which involves "completing a survey about relationship and emotional issues (e.g. depression, domestic violence, stress or worry)." A reminder is mailed out from the practice to all women 14 days following mail-out of the screening survey.

Eligible women will be phoned by a research assistant who will explain the nature of the study (a project looking at ways of improving the care women receive from their GP when they are experiencing relationship and emotional issues, such as being afraid of your partner or ex-partner). It will be explained that the project will involve three surveys over approximately 18 months and that they may or may not be invited to see the GP to discuss relationship and emotional issues depending on the group in which they are placed by chance. Those eligible and agreeing to be involved are sent a baseline survey, information sheet, resource card and a reply paid envelope. Once the baseline survey and consent form have been returned, women are officially enrolled in the trial. A reminder is sent to patients 10 days post baseline survey and a phone call reminder at 20 days. All GPs (and their female patients) in a given wave (there will be four waves) are randomised to intervention or comparison once the cut-off for the return of the baseline survey (30 days following mail-out) for the final GP in the wave has been reached.

### Sequence generation and allocation concealment

Allocation to intervention or comparison will be based on clusters rather than individuals. The trial will be run in four consecutive overlapping waves. Approximately 10 GPs will be randomised in each wave. Characteristics of GPs, including age, sex, years of general practice experience and knowledge about management of partner abuse, will be measured at baseline to check the extent to which randomisation creates equivalence across the two groups. To promote comparability of the intervention and comparison clusters with respect to cluster characteristics, practitioners will be stratified according to whether they are urban or rural and block randomisation with random block sizes will be used within each stratum. The randomisation will be performed by a statistician not directly involved in the study and who is blinded to the identity of the practitioners. Allocation of clusters to intervention or comparison will done following collection of baseline data. In other words, at the time of screening and recruitment of women, the allocation of GPs (and therefore, of women) will be unknown.

### Blinding

**weave **is a pragmatic intervention study. Due to the nature of the intervention (professional training plus patient counselling) it is not possible to blind the GPs to their status as intervention or control. Similarly, the immediate project team is not blind to GP participant status as much interaction between the team and the GPs must occur as part of the training and organising for women to attend their counselling appointments. In the same vein, women are not blinded in that they need to be aware that they may (intervention group) or may not (comparison group) be invited by the GP to discuss relationship issues as part of **weave**. Women will be made aware that they will receive surveys regardless of the group they have been assigned to. There is no blinding as regards data collection based on the CONSORT guidelines [50], as the women and GPs themselves complete the surveys (i.e. data were not collected by a research assistant blinded to the allocation). However the wider investigator team (and the statistician) remain blinded to the identity and allocation of GP participants and women.

### Intervention

The **weave **intervention [37] is a multifaceted, practice-based program refined by the multidisciplinary team and project reference group. It consists of professional, patient and organisational elements. The aim of the professional intervention (**weave **Healthy Relationships Training) is to train GPs in how to respond to IPA when women are identified, and to facilitate GPs to deliver a brief counselling intervention to patients who have been afraid of their partner or ex-partner. It will equip practitioners with an innovative, time-efficient and structured approach to use with patients. The intervention was developed with particular attention to overcome the challenges of changing physician behaviour [53] by being practice-based and including group discussion via teleconference, clinical audits, distance learning, evidence-based guidelines [36] and two interactive practice visits [37]. Key elements of the visits are active listening exercises [54], attitudinal exercises [55], involvement of simulated patients and role play of different readiness for change scenarios [40], use of survivors' voices [56], and modelling of non-abusive behaviours in teaching interactions with health providers [55]. As required, additional practice visits, email and telephone support will be provided.

The patient component of the **weave **intervention involves a brief counselling intervention for delivery by the intervention (trained) group GPs within the primary care setting. Female patients who have been 'afraid' of a partner or ex-partner in the last 12 months (participants in the study) will receive a letter from their GP inviting them to make an appointment to discuss relationship and emotional issues. Women will be offered several 30-minute counselling sessions by their GP for relationship issues and their emotional wellbeing. Where women have not made an appointment within a fortnight of receiving the invitation the research assistant will contact them and offer to connect them immediately with the practice to book an appointment. The main aim of the **weave **brief intervention is to assist women to:

• feel listened to, validated and supported by their GP;

• experience increased awareness about the abuse;

• increase their readiness for change and self-efficacy, and;

• increase their safety planning and behaviours.

At the first visit, the GP establishes with the woman the number of sessions that might be required (up to 6). The woman's readiness for change is established and the GP then selects motivational interviewing and/or problem-solving techniques as part of an appropriate response to the woman's position. GPs complete encounter forms during the women's visits to allow gathering of process data on the content of the counselling.

The minimal organisational change component of the intervention involves circulating information about **weave **to the administrative and clinical staff, placing posters on the wall and working with the practice staff to identify suitable and consistent methods of reminder and recall for the women. Each aspect of the organisational change will prioritise the confidentiality of women and will be practice-centred (i.e. guided by advice of the participating GP and practice manager). At the conclusion of the trial, comparison group GPs will be invited to participate in a day long workshop based on the **weave **Healthy Relationships Training Program.

### Data collection

#### Outcome evaluation

Data will be collected from women by postal questionnaire at the screening stage and at three further points over the duration of the project. Similar to other studies in this area [30,31], we will collect data from women in both groups at baseline, and at 6 and 12 months following the invitation to the intervention group women to attend counselling. Development of study materials has been informed by a primary care cohort study on depression which also gathered data on abuse [51].

#### Screening phase

The primary purpose of the screening questionnaire is to identify women who have been afraid of a partner or ex-partner in the last 12 months and of those, the women willing to be contacted by the project team. The additional items in the survey ask about depression, smoking, alcohol, anxiety, dietary issues and exercise. These help to conceal the purpose of the survey and protect participants. Responses are on a five point likert scale ranging from 'None of the time' to 'All of the time'. If a woman selects an option other than 'None of the time' for the afraid question, and indicates an interest in hearing more about the project, then she is contacted and invited to participate in the trial. Other items include a sub-dimension of the General Practice Assessment Questionnaire [57] and sociodemographic items. We also included items to establish whether women have disclosed being afraid to a GP previously, if they would use help from the GP or general practice nurse if it were available and how comfortable they would be discussing feeling afraid with the GP. It is explained in the screen survey that not every woman who provides her contact details can be contacted by the project team.

#### Trial phase

The primary outcomes (Table 1) include quality of life, measured across four dimensions (physical, psychological, environmental, social) using the WHOQoL-Bref [58], and mental health status, using the mental component of the SF-12 [59]. The third primary outcome is safety and is measured based on the existence of a safety plan (yes/no) and the number of safety behaviours enacted (Safety-Promoting Behaviour Checklist [60]). The secondary outcomes include open ended questions about readiness for change, based on the Domestic Violence Survivor Assessment [61], comfort to discuss abuse with GP (5-point likert scale), GPs' inquiry about the safety of women and their children (yes/no) and anxiety and depression, based on the Hospital Anxiety and Depression Scale [62]. Health care utilisation is measured based on visits to health professionals, days out-of-role and hospital admissions. Other variables measured at different stages of the trial were included to investigate mediating variables (see Table 2) and to provide process data to help understand why the intervention may or may not have been effective. Harm associated with participation in the research (e.g. acceptability of screening, distress caused by being invited into the project, partner's awareness of the research, adverse effects arising from participating in counselling, response burden) was measured using an adapted version of the COST questionnaire [31].

**Table 2:** Other variables and measures included in 6 and 12 month surveys

Variable	Measure/tool
Sense of safety	How safe have you felt at home in the last two weeks/6/12 months ago? (visual analogue scale)
Safety behaviours	What things do you do (or have you done in the last 6 months) to keep you safe from your partner or ex-partner? (open-ended question)
Nature/frequency of abuse	Composite Abuse Scale [7]
Health status	Short Form-12 (PCS) [59] Smoking Alcohol (AUDIT) [69] Medications (analgesics, antidepressants and sedatives)
Post-traumatic stress disorder	Short Screening Scale for DSM-IV PTSD [70]
Self-esteem and self-efficacy	Rosenberg Self-Esteem Scale [71] Generalized Self-Efficacy Scale [72]
Social support	Oslo 3 Social Support [73]

The readiness of GPs to manage intimate partner abuse is assessed before and after the training using PREMIS, a validated questionnaire assessing knowledge, attitude and behaviours of doctors with regard to IPA [63].

#### Economic evaluation

The primary economic evaluation will use a cost-consequences analysis, with any incremental costs compared to all incremental outcomes as detailed above. If this does not reveal a dominant result for the cost-effectiveness of the intervention, secondary economic analysis will involve incremental cost-effectiveness analysis using individual outcome measures and cost-utility analysis using SF-12 data. The economic evaluation will be conducted from both a health care and societal perspective, with costs including resources used in intervention delivery and practice-based system change and women's use of health care and other societal resources.

#### Process evaluation

**Figure 2:**
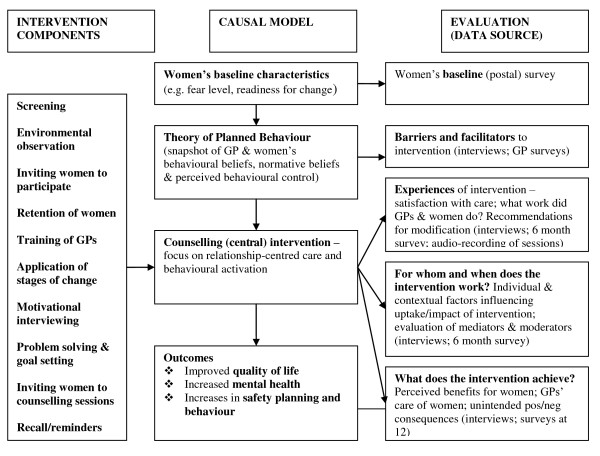
**weave causal model**.

The Realistic Evaluation model was used in the trial to develop a causal model (Figure 2) to allow understanding of 'what works for whom in what circumstances?' [64]. This evaluative framework examines context, mechanism and outcomes. Process data will include completion of encounter forms describing what GPs did during the sessions e.g. counselling methods used, billing, follow-up and written plans. In addition we will ask GPs to audio-record consultations. We anticipate that only a small proportion of GPs and women will agree to have the sessions recorded. We will conduct semi-structured interviews with a sample of up to 20 women from each arm of the trial after the 12 month assessment. We will purposively sample the women such that a range of women's level of fear, severity of abuse and their readiness to change at baseline are represented. The purpose will be to assess their experiences of receiving the intervention or usual care and perceived outcomes. We will gather data on satisfaction with counselling sessions/usual care, extent to which expectations of sessions/usual care were met, changes to usual GP care, quality of relationship with GP, experiences of being in the project, relationship with research team, and any changes women made in their relationships as a result of being involved in **weave**. Individual semi-structured telephone interviews with all GPs from both comparison and intervention at the end of the trial will assess their perceptions of the research and intervention process and the impact on their practice, both positive and negative. We will ask about satisfaction with training and counselling process/usual care, perceived impact of counselling/usual care on women, whether expectations of being involved in **weave **were met and perceived impact on their practice and sustainability of skills and practice. Data from all sources including the 6 month patient surveys will be combined to understand what works for whom in what circumstances.

### Data analysis and reporting

Characteristics of GPs and women will be summarised using frequencies and percentages for categorical data, and means and standard deviations or percentiles for continuous data, for the two study arms. GP and women's characteristics will be compared between the two arms at recruitment to ensure that randomisation was effective. Intra-cluster correlations will be calculated for key outcome variables and patient variables at baseline. Appropriate modelling techniques will be used to account for the complexity of the study design, its hierarchical structure (women clustered within practices), stratification of practices at randomisation and repeated measures over time. GP practice will be set as the primary sampling unit and analysis will be intention-to-treat. Marginal logistic regression using Generalised Estimating Equations (GEE) with information sandwich estimates of standard error will be used for the binary outcomes. Mixed-effects linear regression will be used to compare the scores between the two study groups for mental health status and quality of life measures. Baseline outcome measures and any imbalanced factors strongly associated with the outcomes will be adjusted for in the regression model. An independent data monitoring committee (DMC) consisting of 5 members (GP/researcher, IPA researcher, community service worker, GP, and a statistician) will be convened on approximately four occasions over the course of the trial. The aim of the **weave **DMC is to monitor the safety of the participants and ensure the integrity of the trial data. This will be achieved by checking interim data and monitoring progress against the trial protocol including recruitment rates, uptake of the intervention and loss to follow-up.

## Discussion

In summary, there is a strong rationale for developing and testing interventions of screening and counselling for women who experience partner abuse, for embedding this research in primary care and for measuring the effect in terms of health outcomes for women. Primary care allows considerable scope in terms of the women who are reached, and is unique in that it has the potential to facilitate early intervention as well as support for women who are in recovery but remain at risk. Notwithstanding the challenges [65], well-designed randomised controlled trials are essential for testing hypotheses with strong theoretical underpinnings to produce high quality evidence [30]. Evaluation needs to incorporate adequate follow-up and a focus on safety and health outcomes for women. Finally, process measurement is essential to explain the 'why and how' of the intervention, focusing on areas such as uptake of intervention, harm, readiness for change, inquiry by health professionals, abuse, support, self-efficacy and expectations. With intimate partner abuse the leading contributor to death, disability and illness among Victorian women aged 15 to 44 years [10], there is an urgent need to build evidence about effective response to this complex social problem in primary care. An 'effective' response not only requires an assessment of the safety of women and children, it must also respect and promote the dignity of women, validate and understand the diversity of women's experiences, withhold judgement about what a woman should do and when, and place ongoing support at the centre of the interaction between the woman and practitioner.

## Competing interests

The authors declare that they have no competing interests.

## Authors' contributions

KH has major responsibility for the design and conduct of the **weave **trial, co-developed and delivers the GP training and drafted and revised this manuscript. JG contributed to the design of **weave **and the development of the training. LOD provides substantial input to the implementation of **weave **and drafting and revising of the manuscript. AT, GF, JA and SB contributed to the design of **weave **and the drafting and revising of the manuscript. PC advises on design and analysis and contributed to the draft manuscript. All authors approved the final manuscript.

## Pre-publication history

The pre-publication history for this paper can be accessed here:

http://www.biomedcentral.com/1471-2458/10/2/prepub

## References

[B1] Krug EG Mercy JA Dahlberg LL Zwi AB The world report on violence and health Lancet 2002 360 9339 1083 1088 10.1016/S0140-6736(02)11133-0 12384003

[B2] Heise L Violence against women: an integrated, ecological framework Violence Against Women 1998 4 3 262 290 10.1177/1077801298004003002 12296014

[B3] Tjaden P Thoennes N Extent, Nature, and Consequences of Intimate Partner Violence 2000 Washington: US Dept of Justice 1 62

[B4] Campbell JC Health consequences of intimate partner violence Lancet 2002 359 1331 1336 10.1016/S0140-6736(02)08336-8 11965295

[B5] Garcia-Moreno C Jansen HA Ellsberg M Heise L Watts CH Prevalence of intimate partner violence: findings from the WHO multi-country study on women's health and domestic violence Lancet 2006 368 9543 1260 1269 10.1016/S0140-6736(06)69523-8 17027732

[B6] Hegarty K Bush R Prevalence of partner abuse in women attending Australian general practice: A cross-sectional survey Aust N Z J Public Health 2002 26 5 437 442 10.1111/j.1467-842X.2002.tb00344.x 12413288

[B7] Hegarty K Bush R Sheehan M The composite abuse scale: further development and assessment of reliability and validity of a multidimensional partner abuse measure in clinical settings Violence Vict 2005 20 5 529 547 10.1891/vivi.2005.20.5.529 16248489

[B8] Hegarty K Gunn J Chondros P Small R Association of depression and partner abuse in women attending general practice: A cross sectional survey BMJ 2004 328 7440 621 624 10.1136/bmj.328.7440.621 15016694 PMC381136

[B9] Richardson J Coid J Petruckevitch A Wai Shan C Moorey S Feder G Identifying domestic violence: cross sectional study in primary care BMJ 2002 324 1 6 10.1136/bmj.324.7332.274 11823360 PMC65060

[B10] VicHealth The health costs of violence. Measuring the burden of diseases caused by intimate partner violence Melbourne 2005

[B11] Golding J Intimate partner violence as a risk factor for mental disorders: a meta-analysis J Fam Viol 1999 14 2 99 132 10.1023/A:1022079418229

[B12] Coid J Yang M Roberts A Ullrich S Moran P Bebbington P Brugha T Jenkins R Farrell M Lewis G Violence and psychiatric morbidity in the national household population of Britain: public health implications Br J Psychiatry 2006 189 12 19 10.1192/bjp.189.1.12 16816300

[B13] Heise L Ellsberg M Gottmoeller M A global overview of gender-based violence Int J Gynaecol Obstet 2002 78 Suppl 1 S5 14 10.1016/S0020-7292(02)00038-3 12429433

[B14] Ellsberg M Jansen HA Heise L Watts CH Garcia-Moreno C Intimate partner violence and women's physical and mental health in the WHO multi-country study on women's health and domestic violence: an observational study Lancet 2008 371 9619 1165 1172 10.1016/S0140-6736(08)60522-X 18395577

[B15] Martin SL Mackie L Kupper LL Buescher PA Moracco KE Physical abuse of women before, during, and after pregnancy JAMA 2001 285 12 1581 1584 10.1001/jama.285.12.1581 11268265

[B16] Murphy CC Schei B Myhr TL Du Mont J Abuse: a risk factor for low birth weight? A systematic review and meta-analysis CMAJ 2001 164 11 1567 1572 11402794 PMC81110

[B17] Sharps PW Laughon K Giangrande SK Intimate partner violence and the childbearing year: maternal and infant health consequences Trauma Violence Abuse 2007 8 2 105 116 10.1177/1524838007302594 17545568

[B18] Boy A Salihu HM Intimate partner violence and birth outcomes: a systematic review Int J Fertil Womens Med 2004 49 4 159 164 15481481

[B19] Silverman JG Decker MR Reed E Raj A Intimate partner violence victimization prior to and during pregnancy among women residing in 26 U.S. states: associations with maternal and neonatal health Am J Obstet Gynecol 2006 195 1 140 148 10.1016/j.ajog.2005.12.052 16813751

[B20] Brown SJ McDonald EA Krastev AH Fear of an intimate partner and women's health in early pregnancy: findings from the Maternal Health Study Birth 2008 35 4 293 302 10.1111/j.1523-536X.2008.00256.x 19036042

[B21] Kitzmann KM Gaylord NK Holt AR Kenny ED Child witnesses to domestic violence: A meta-analytic review J Consult Clin Psychol 2003 71 2 339 352 10.1037/0022-006X.71.2.339 12699028

[B22] Plichta SB Interactions between victims of intimate partner violence against women and the health care system: policy and practice implications Trauma Violence Abuse 2007 8 2 226 239 10.1177/1524838007301220 17545576

[B23] Plichta S The effects of woman abuse on health care utilization and health status: a literature review Womens Health Issues 1992 2 3 154 163 10.1016/S1049-3867(05)80264-6 1422244

[B24] Hegarty KL Taft AJ Overcoming the barriers to disclosure and inquiry of partner abuse for women attending general practice Aust N Z J Public Health 2001 25 5 433 437 11688623

[B25] Bradley F Smith M Long J O'Dowd T Reported frequency of domestic violence: cross sectional survey of women attending general practice BMJ 2002 324 7332 271 10.1136/bmj.324.7332.271 11823359 PMC65059

[B26] Burge SK Schneider FD Ivy L Catala S Patients' advice to physicians about intervening in family conflict Ann Fam Med 2005 3 3 248 254 10.1370/afm.287 15928229 PMC1466879

[B27] Feder G Hutson M Ramsay J Taket A Women exposed to intimate partner violence: expectations and experiences when they encounter health care professionals: a meta-analysis of qualitative studies Arch Intern Med 2006 166 1 22 37 10.1001/archinte.166.1.22 16401807

[B28] Gutmanis I Beynon C Tutty L Wathen CN MacMillan HL Factors influencing identification of and response to intimate partner violence: a survey of physicians and nurses BMC Public Health 2007 7 12 10.1186/1471-2458-7-12 17250771 PMC1796870

[B29] Taft A Broom D Legge D General practitioner management of intimate partner abuse and the whole family: a qualitative study BMJ 2004 328 618 621 10.1136/bmj.38014.627535.0B 14766719 PMC381135

[B30] Feder G Ramsay J Dunne D Rose M Arsene C Norman R How far does screening women for domestic (partner) violence in different health-care settings meeting the UK National Screening Committee criteria for a screening programme in terms of condition, screening method and intervention? Systematic reviews of nine UK National Screening Committee criteria Health Technol Assess 2009 13 16 1 113 10.3310/hta13160 19272272

[B31] MacMillan HL Wathen CN Jamieson E Boyle MH Shannon HS Ford-Gilboe M Worster A Lent B Coben JH Campbell JC Screening for intimate partner violence in health care settings: a randomized trial JAMA 2009 302 5 493 501 10.1001/jama.2009.1089 19654384

[B32] Moracco KE Cole TB Preventing intimate partner violence: screening is not enough JAMA 2009 302 5 568 570 10.1001/jama.2009.1135 19654392

[B33] Straus MA Straus MA, Gelles RJ The Conflict Tactics Scale and its critics: An evaluation and new data on validity and reliability Physical Violence in American Families Risk Factors and Adaptions to Violence in 8,145 Families 1990 New Brunswick: Transaction Publishers 49 75

[B34] Sohal H Eldridge S Feder G The sensitivity and specificity of four questions (HARK) to identify intimate partner violence: a diagnostic accuracy study in general practice BMC Fam Pract 2007 8 49 10.1186/1471-2296-8-49 17727730 PMC2034562

[B35] Ramsay J Feder G Rivas C Interventions to reduce violence and promote the physical and psychosocial well-being of women who experience partner abuse: a systematic review 2006 London: UK Department of Health http://www.dh.gov.uk/en/Publicationsandstatistics/Publications/PublicationsPolicyAndGuidance/DH_4126266

[B36] Taft AJ Hegarty KL Feder GS Tackling partner violence in families Med J Aust 2006 185 10 535 536 17115963 10.5694/j.1326-5377.2006.tb00686.x

[B37] Hegarty K O'Doherty L Gunn J Pierce D Taft A A brief counseling intervention by health professionals utilising the 'readiness to change' concept for women experiencing intimate partner abuse: The weave project J Fam Studies 2008 14 2-3 376 388

[B38] Prochaska J Clemente C Stages of change in the modification of problem behaviors Prog Behav Modif 1992 28 183 218 1620663

[B39] Zink T Elder N Jacobson J Klostermann B Medical management of intimate partner violence considering the stages of change: precontemplation and contemplation Ann Fam Med 2004 2 3 231 239 10.1370/afm.74 15209200 PMC1466661

[B40] Frasier PY Slatt L Kowlowitz V Glowa PT Using the stages of change model to counsel victims of intimate partner violence Patient Educ Couns 2001 43 2 211 217 10.1016/S0738-3991(00)00152-X 11369155

[B41] Cluss PA Chang JC Hawker L Scholle SH Dado D Buranosky R Goldstrohm S The process of change for victims of intimate partner violence: support for a psychosocial readiness model Womens Health Issues 2006 16 5 262 274 10.1016/j.whi.2006.06.006 17055379

[B42] Miller WR Rollncik S Motivational Interviewing: Preparing people for change 2002 2 New York: Guilford Press

[B43] Mynors-Wallis LM Gath DH Day A Baker F Randomised controlled trial of problem solving treatment, antidepressant medication, and combined treatment for major depression in primary care BMJ 2000 320 7226 26 30 10.1136/bmj.320.7226.26 10617523 PMC27250

[B44] Rice V Stead L Nursing interventions for smoking cessation Cochrane Database Syst Rev 2004 CD001188 14973964 10.1002/14651858.CD001188.pub2

[B45] McFarlane J Groff J O'Brien J Watson K Secondary prevention of intimate partner violence: a randomized controlled trial Nurs Res 2006 55 1 52 61 10.1097/00006199-200601000-00007 16439929

[B46] McFarlane J Campbell JC Sharps P Watson K Abuse during pregnancy and femicide: urgent implications for women's health Obstet Gynecol 2002 100 1 27 36 10.1016/S0029-7844(02)02054-9 12100800

[B47] Tiwari A Leung W Leung TY Humphreys J Parker B Ho P A randomised controlled trial of empowerment training for Chinese abused pregnant women in Hong Kong BJOG 2005 112 1249 1256 10.1111/j.1471-0528.2005.00709.x 16101604

[B48] Sullivan CM Bybee DI Reducing violence using community-based advocacy for women with abusive partners J Consult Clin Psychol 1999 67 1 43 53 10.1037/0022-006X.67.1.43 10028208

[B49] Wathen N HL M Interventions for Violence Against Women: Scientific Review JAMA 2003 289 589 600 10.1001/jama.289.5.589 12578492

[B50] Campbell MK Elbourne DR Altman DG CONSORT statement: extension to cluster randomised trials BMJ 2004 328 7441 702 708 10.1136/bmj.328.7441.702 15031246 PMC381234

[B51] Gunn JM Gilchrist GP Chondros P Ramp M Hegarty KL Blashki GA Pond DC Kyrios M Herrman HE Who is identified when screening for depression is undertaken in general practice? Baseline findings from the Diagnosis, Management and Outcomes of Depression in Primary Care (diamond) longitudinal study Med J Aust 2008 188 12 Suppl S119 125 18558911 10.5694/j.1326-5377.2008.tb01874.x

[B52] Taft AJ Small R Hegarty KL Lumley J Watson LF Gold L MOSAIC (MOthers' Advocates In the Community): protocol and sample description of a cluster randomised trial of mentor mother support to reduce intimate partner violence among pregnant or recent mothers BMC Public Health 2009 9 159 10.1186/1471-2458-9-159 19473534 PMC2702379

[B53] Davis DA Thomson MA Oxman AD Haynes RB Changing physician performance. A systematic review of the effect of continuing medical education strategies JAMA 1995 274 9 700 705 10.1001/jama.274.9.700 7650822

[B54] Gunn J Hegarty K Nagle C Forster D Brown S Lumley J Putting woman-centred care into practice: a new (ANEW) approach to psychosocial risk assessment during pregnancy Birth 2006 33 1 46 55 16499531 10.1111/j.0730-7659.2006.00073.x

[B55] Warshaw C Roberts G, Hegarty K, Feder G Educating health professionals: changing attitudes and overcoming barriers Intimate partner abuse and health professionals: New approaches to domestic violence 2006 London: Elsevier

[B56] Short L Johnson D Osattin A Recommended components of health care provider training programs on intimate partner violence Am J of Prev Med 1998 14 4 283 288 10.1016/S0749-3797(98)00007-5 9635072

[B57] Mead N Bower P Roland M The General Practice Assessment Questionnaire (GPAQ) - development and psychometric characteristics BMC Fam Pract 2008 9 13 10.1186/1471-2296-9-13 18289385 PMC2277420

[B58] Skevington SM Lotfy M O'Connell KA The World Health Organization's WHOQOL-BREF quality of life assessment: psychometric properties and results of the international field trial. A report from the WHOQOL group Qual Life Res 2004 13 2 299 310 10.1023/B:QURE.0000018486.91360.00 15085902

[B59] Ware J Jr Kosinski M Keller SD A 12-Item Short-Form Health Survey: construction of scales and preliminary tests of reliability and validity Med Care 1996 34 3 220 233 10.1097/00005650-199603000-00003 8628042

[B60] McFarlane J Parker B Soeken K Silva C Reel S Safety behaviors of abused women after an intervention during pregnancy J Obstet Gynecol Neonatal Nurs 1998 27 1 64 69 10.1111/j.1552-6909.1998.tb02592.x 9475129

[B61] Dienemann J Campbell J Landenburger K Curry MA The domestic violence survivor assessment: a tool for counseling women in intimate partner violence relationships Patient Educ Couns 2002 46 3 221 228 10.1016/S0738-3991(01)00216-6 11932120

[B62] Zigmond AS Snaith RP The hospital anxiety and depression scale Acta Psychiatr Scand 1983 67 6 361 370 10.1111/j.1600-0447.1983.tb09716.x 6880820

[B63] Short LM Alpert E Harris J Surprenant Z A tool for measuring physician readiness to manage intimate partner violence Am J Prev Med 2006 30 2 173 180 10.1016/j.amepre.2005.10.009 16459217 PMC1451776

[B64] Pawson R Tilley N Realistic Evaluation 1997 London: SAGE Publications Ltd

[B65] Spangaro J Zwi AB Poulos R The elusive search for definitive evidence on routine screening for intimate partner violence Trauma Violence Abuse 2009 10 1 55 68 10.1177/1524838008327261 19056688

[B66] Hawthorne G Herrman H Murphy B Interpreting the WHOQOL-Bref: preliminary population norms and effect sizes Soc Indicators Res 2006 77 37 59 10.1007/s11205-005-5552-1

[B67] Wittenberg E Lichter EL Ganz ML McCloskey LA Community preferences for health states associated with intimate partner violence Med Care 2006 44 8 738 744 10.1097/01.mlr.0000215860.58954.86 16862035

[B68] McFarlane J Malecha A Gist J Watson K Batten E Hall I Smith S Increasing the safety-promoting behaviors of abused women Am J Nurs 2004 104 3 40 50 quiz 50-41. 15108570 10.1097/00000446-200403000-00019

[B69] Saunders JB Asland OG Babor TF de la Fuente JR Grant M Development of the Alcohol Use Disorders Identification Test (AUDIT): WHO Collaborative Project on Early Detection of Persons with Harmful Alcohol Consumption--II Addiction (Abingdon, England) 1993 88 6 791 804 8329970 10.1111/j.1360-0443.1993.tb02093.x

[B70] Breslau N Peterson EL Kessler RC Schultz LR Short screening scale for DSM-IV posttraumatic stress disorder Am J Psychiatry 1999 156 6 908 911 10360131 10.1176/ajp.156.6.908

[B71] Rosenberg M Society and the adolescent self-image 1965 Princeton, NJ: Princeton University Press

[B72] Schwarzer R Jerusalem M Weinman J, Wright S, Johnston M Generalized Self-Efficacy scale Measures in health psychology: A user's portfolio. Causal and control beliefs 1995 Windsor, England: NFER-NELSON 35 37

[B73] Meltzer H Nosikov A, Gudex C Development of a common instrument for mental health EUROHIS: developing common instruments for health surveys 2003 Amsterdam: IOS Press

